# On the Quest of Cellular Functions of PEA-15 and the Therapeutic Opportunities

**DOI:** 10.3390/ph8030455

**Published:** 2015-07-31

**Authors:** Yufeng Wei

**Affiliations:** 1 Department of Chemistry, New Jersery City University, 2039 Kennedy Blvd, Jersey City, NJ 07305, USA; E-Mail: Yufeng.Wei@shu.edu; Tel.: +1-973-275-2335; Fax: +1-973-275-2489; 2 Institute of NeuroImmune Pharmacology, Seton Hall University, 400 South Orange Ave, South Orange, NJ 07094, USA

**Keywords:** PEA-15, MAP kinase, apoptosis, phosphorylation, protein-protein interaction

## Abstract

Phosphoprotein enriched in astrocytes, 15 KDa (PEA-15), a ubiquitously expressed small protein in all mammals, is known for decades for its potent interactions with various protein partners along distinct biological pathways. Most notable interacting partners of PEA-15 include extracellular signal-regulated kinase 1 and 2 (ERK1/2) in the mitogen activated protein kinase (MAPK) pathway, the Fas-associated death domain (FADD) protein involving in the formation of the death-inducing signaling complex (DISC), and the phospholipase D1 (PLD1) affecting the insulin sensitivity. However, the actual cellular functions of PEA-15 are still mysterious, and the question why this protein is expressed in almost all cell and tissue types remains unanswered. Here we synthesize the most recent structural, biological, and clinical studies on PEA-15 with emphases on its anti-apoptotic, anti-proliferative, and anti-inflammative properties, and propose a converged protective role of PEA-15 that maintains the balance of death and survival in different cell types. Under conditions that this delicate balance is unsustainable, PEA-15 may become pathological and lead to various diseases, including cancers and diabetes. Targeting PEA-15 interactions, or the use of PEA-15 protein as therapeutics, may provide a wider window of opportunities to treat these diseases.

## 1. Introduction

When PEA-15 (phosphoprotein enriched in astrocytes, 15 KDa) was first discovered two decades ago, it was recognized as an endogenous substrate of protein kinase C (PKC) and highly enriched in the astrocytes [1]. PEA-15 was later found to be expressed more ubiquitously among various tissues and species [2,3], with two phosphorylation sites: Ser^104^, located within the motif ^99^LTRIPSAKK^107^, is the target of PKC; and Ser^116^, located within the motif ^111^DIRQPSEEEIIK^122^, is the target of calcium/calmodulin-dependent protein kinase II (CaMKII) [4] and protein kinase B/Akt [5]. However, PEA-15 protein did not gain much traction among the research community until it was identified to affect and regulate several critical cellular pathways that could lead to major diseases, such as cancers and type 2 diabetes. PEA-15 was identified to be overexpressed in fibroblasts, skeletal muscle, and adipose tissues in type 2 diabetes, and accordingly named as PED (phosphoprotein enriched in diabetes) [6]. PED/PEA-15 inhibits membrane association of insulin-sensitive glucose transporter 4 (GLUT4) through the interaction with phospholipase D1 and D2 (PLD1/2) [7], which activates PKC-α and -β, and blocks insulin-induction PKC-ζ activity [8]. PED/PEA-15 overexpression is considered a common defect in first-degree relatives of type 2 diabetic patients, and is associated with reduced insulin sensitivity in these individuals [9]. Later, PEA-15 was found to inhibit Fas/TNF (tumor necrosis factor)-α induced apoptosis by binding to the adapter protein FADD (Fas-associated death domain) and blocking the recruitment and activation of caspase-8 [10,11,12]. Moreover, PEA-15 was reported to activate extracellular signal-regulated kinase 1 and 2 (ERK1/2) in the mitogen-activated protein (MAP) kinase pathway in a Ras-dependent manner [13]. PEA-15 binds to ERK, promoting cytosolic localization of ERK and blocking ERK-dependent transcription and proliferation [14]. PEA-15 binding to ERK1/2 also blocks plasma membrane association of ERK and prevents threonine phosphorylation of fibroblast receptor substrate 2α (FRS2α). This action prolongs fibroblast growth factor (FGF)-induced tyrosine phosphorylation of FRS2α that sustains MEK and ERK activation, while inhibits the transcriptional activities of ERK1/2 [15]. PEA-15 binds to p90 ribosomal S6 kinase isozyme 2 (RSK2), but not RSK1 [16], and it enhances RSK2 activation by ERK [17]. These interactions put PEA-15 in the crossroad of several vital biological pathways, making PEA-15 clinically relevant in cancer therapies and a diabetes biomarker. The roles of PEA-15 in cancer development and progression are complex and controversial [18].

Structurally, PEA-15 possesses an N-terminal death effector domain (DED), consisting of residues 1-90, and a long, flexible C-terminal tail, consisting of residues 91-130 [19]. DED, together with death domain (DD), caspase activation and recruitment domain (CARD), and pyrin domain (PYD), comprises of the superfamily of death structural domain, characterized by a canonical six-helix bundle fold [20,21]. PEA-15 contains the characteristic surface feature of DED, termed charge triad, of residues D^19^-R^72^xD^74^L, forming a relatively strong charged hydrogen bonding network [22]. The N-terminus of the DED also possesses a leucine-rich nuclear exporting sequence (NES), ^7^LLQDLTNNITL^17^, which promotes nuclear export of ERK and cytosolic accumulation [14]. PEA-15 has no catalytic activities, and all its functions are exerted through protein-protein interactions [23]. PEA-15 appears to adopt different surfaces to interact with its binding partners. PEA-15 interacts with FADD DED using a surface patch adjacent to helix α2 [24]; it utilizes both DED residues on helices α1, α5 and α6 and C-terminal tail residues to bind to ERK2 [25], while its conformation at helices α2, α3, and α4 undergoes significant changes [26,27]; it binds to RSK2 with its C-terminus but not the N-terminal DED [16]; and it likely utilizes its first 24 residues to interact with PLD1 C-terminal D4 domain [28,29]. PEA-15 has been recognized as a key multi-protein binding molecule modulating a number of cellular processes, including proliferation, apoptosis, and glucose metabolism [23].

Although the involvement of PEA-15 in insulin resistance, anti-apoptosis, and cell cycle modulation has been widely demonstrated, the basic cellular functions and roles of PEA-15 and its ubiquitous expression in almost all cell and tissue types are still largely mysterious. Based on most recent findings by us and other researchers in the field, we hypothesize that PEA-15 performs protective roles to maintain a delicate balance between apoptosis and proliferation through controlled distribution of phosphorylated and unphosphorylated components, ensuring proper levels of activation of various cellular pathways. Under conditions that the balance is unsustainable, such as fluctuation of kinase and phosphatase compositions and concentrations in the cells, PEA-15 may go from protective to pathogenic, inducing diseases including diabetes and cancers. The underlying mechanisms involving PEA-15 under various conditions will be specifically discussed.

## 2. Protective Roles of PEA-15 and Modulations of PEA-15 Functions

### 2.1. PEA-15 Protects Mature Neurons from Fas/TNFα-Induced Apoptosis

Mature neurons are specialized cells in the CNS that permanently remain in a quiescent stage, or G_0_ phase, without proliferation. It is, therefore, critically important that neurons do not undergo apoptosis as the dead cells cannot be replaced. Excessive nerve cell death could lead to severe neurodegenerative diseases, including amyotrophic lateral sclerosis (ALS), Parkinson’s, Alzheimer’s, and Huntington’s diseases [30]. Widespread expression of PEA-15 was found throughout the normal adult brain [31]. As a member of the DED subfamily within the death domain superfamily, PEA-15 involves in the homotypic interactions with other DED-containing proteins, specifically, FADD (Fas-associated death domain) and procaspase-8, inhibiting death signaling [20,21]. Using PEA-15 null (PEA-15^−/−^) mice, which appear healthy and fertile comparing to wild-type control C57BL/6 animals, it was shown that astrocytes not expressing PEA-15 undergo apoptosis within 24 hours of TNFα treatment, while the wild-type cells do not show major morphological changes under the same condition [11], indicating the protective roles of PEA-15 in the CNS. A recent study showed that PEA-15 is expressed at higher levels in the neocortices from six-month old TgCRND8 mice, a human Alzheimer’s disease (AD) transgenic mouse model, as compared to age-matched non-transgenic mice, and PEA-15 expressing reactive astrocytes are associated with neocortical amyloid plaques in TgCRND8 mice and in postmortem human AD brains [32]. The high expression of PEA-15 in plaque-associated reactive astrocytes is speculated to protect the astrocytes from apoptosis induced by elevated TNFα level in the vicinity of the amyloid plaques [32]. The neuroprotective effects of PEA-15 were also demonstrated in a Parkinson’s disease (PD) mouse model, where PEA-15 was fused to a cell permeable protein transduction domain (PTD), PEP-1, and transduced into SH-SY5Y neuroblastoma and BV2 microglia cells, as well as substantia nigra (SN) brain section of PD mouse model. Transduced PEP-1-PEA-15 protects against MPP^+^ (1-methyl-4-phenylpyridinium)-induced neurotoxicity and neuronal cell death in cell viability assay, and prevents dopaminergic neuronal cell death in a chronic MPTP (1-methyl-4-phenyl-1,2,3,6-tetrahydropyridine)-induced PD mouse model [33]. In addition, treatment with PEP-1-PEA-15 ameliorates MPTP-induced behavioral dysfunctions and increased dopamine levels in the striatum [33].

However, this anti-apoptotic property of PEA-15 is blamed for the resistance to chemotherapy in various cancers. In breast cancer cell line MCF-7 and HeLa cells, PEA-15 overexpression inhibits Fas ligand (FasL) and Fas/TNFα apoptotic effects by blocking the interaction between FADD and caspase-8, preventing the recruitment and activation of caspase-8 at the death-inducing signaling complex (DISC) [10]. PEA-15 was also reported to protect malignant glioma cells from TNF-related apoptosis-inducing ligand (TRAIL) mediated apoptosis [12], and TRAIL resistant effect depends on both PKC [12] and CaMKII activities [34], suggesting doubly-phosphorylated PEA-15 is responsible for the anti-apoptotic effect of PEA-15. In human breast cancer, PEA-15 is overexpressed in correlation with Akt up-regulation, which was considered to contribute to the resistance to breast cancer cell death [35]. PEA-15 was also identified to express strongly in various lung tumor types, and the high level expression of PEA-15 in non-small cell lung cancer (NSCLC) cell line is correlated with the resistance of TRAIL-induced cancer cell death [36].

### 2.2. PEA-15 Protects Tissues from Malignant Cell Growth

MAP kinase signaling pathway is central in regulating cell proliferation, cell differentiation, and apoptosis, and the localization of ERK1/2 essentially determines the fate of cells [37]. By increasing cytoplasmic localization of activated ERK1/2, PEA-15 prevents tumor cell invasion and proliferation [38], inhibits tumorigenesis in triple-negative breast cancer [39], and is associated with prolonged overall survival by inducing autophagy in human ovarian cancer cell [40]. In a similar fashion, PEA-15 inhibits fibroblast motility and wound closure in an ERK1/2-dependent mechanism [41]. PEA-15 also regulates JNK (c-Jun N-terminal Kinase) signaling to promote autophagy in glioma cells [42]. The antitumor activity of E1A in ovarian cancer is associated with PEA-15 translocalization of ERK from nuclear to cytoplasm [43]. PEA-15 modulates coxsackievirus–adenovirus Receptor (CAR) expression and adenoviral infectivity via ERK-mediated signals in glioma cells, representing PEA-15 as a predictive marker in glioblastoma [44]. PEA-15 expression levels were found to be inversely associated with malignancy grade of astrocytic tumors, and high PEA-15 expression was correlated to longer overall survival [45]. In colorectal carcinoma (CRC), the expression of PEA-15 was reported to be significantly associated with pathological T (pT) stadium, which is defined by the extent of tumor invasion into the colonic wall, suggesting a negative relationship between PEA-15 expression and grade of malignancy [46]. Increased PEA-15 expression strongly inhibits clonogenicity, proliferation, and invasiveness of CRC cells, while as the same time, significantly protects CRC cells from apoptosis by cytotoxic drugs, by death ligand TRAIL, or by serum withdrawal [46].

Most recently, PEA-15 was associated with cell cycle checkpoints and cell cycle arrest in malignant cells. PEA-15 was reported to play a role in promoting DNA damage-induced G_2_/M checkpoint. DNA damage stabilizes PEA-15 by preventing polyubiquitination and proteasome-mediated degradation [47], similar to the mechanism of DNA damage-induced upregulation of other tumor suppressor proteins, such as p53 [48]. PEA-15 protein levels oscillate throughout the cell cycle with peak expression during G_2_/M, and PEA-15 knockdown results in G_2_/M checkpoint defect due to increased activation of CDC25C by ERK1/2, which subsequently elevates cyclin-dependent kinase CDK1/cyclin B activity [47]. PEA-15 controls cell cycle progression by inhibiting ERK-dependent, c-JUN-mediated transcriptional activation of CDK6, and regulates RAS-mediated neoplastic transformation by suppressing CDK6 activity [47]. PEA-15 was also found to be epigenetically silenced through promotor DNA hypermethylation in colorectal, lung, and breast cancer tissues [47]. Another study showed that in human diploid fibroblast (HDF) senescent cells, PEA-15 knockdown significantly progressed G_1_ arrested cells to S-phase through nuclear translocation of ERK1/2, and activated cell proliferation [49]. These data suggested an important role of PEA-15 in maintaining genome integrity by regulating cell cycles and promoting checkpoints. PEA-15 knockdown could lead to increase mutations in genomic DNA with accelerated cell cycle.

### 2.3. PEA-15 Prevents Tissue Damage from Excessive Inflammation

T lymphocyte activation is a vital step in initiating immune responses. Using PEA-15 null (PEA-15^−/−^) mouse model, it has been demonstrated that PEA-15 can affect T-cell activation but not apoptosis [50]. PEA-15-deficient T cells are hyperproliferative due to unrestricted ERK1/2 activation and nuclear localization, resulting in elevated IL (Interleukin)-2 secretion and transcription. The excessive production of inflammatory cytokines, such as IL-2, could cause severe tissue damages. PEA-15 is hence considered as a critical regulatory protein to control proper level of T-cell activation and inflammation [50].

## 3. PEA-15 Phosphorylation Homeostasis

**Figure 1:**
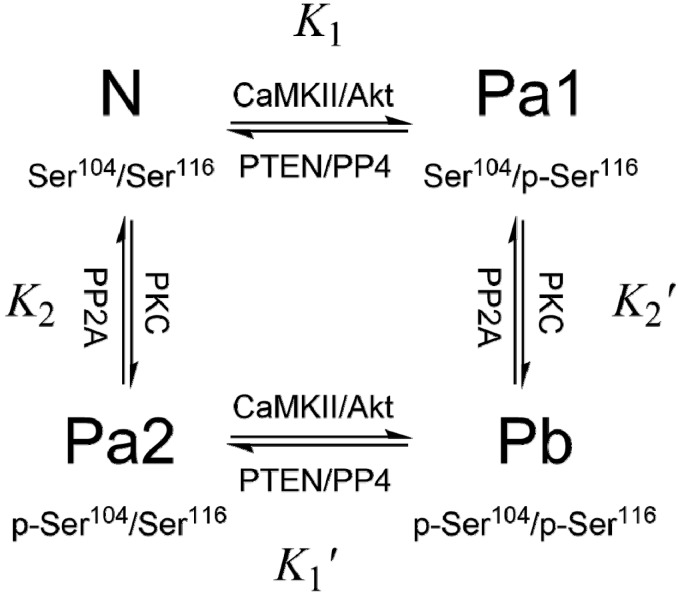
PEA-15 phosphorylation cycle. Ser^104^ is phosphorylated by PKC, and Ser^116^ is phosphorylated by CaMKII or PKB/Akt. PP2A is indicated to dephosphorylate p-Ser^104^, while PP4 is associated with dephosphorylation at p-Ser^116^. PTEN reduces p-Ser^116^ content by deactivating Akt. The relative protein kinase/phosphatase activities mediate PEA-15 phosphorylation homeostasis.

The expression of PEA-15 is tightly controlled by both positive and negative regulatory factors that regulate lipid and glucose homeostasis: hepatocyte nuclear factor 4α (HNF-4α) represses PEA-15 expression, while chicken ovalbumin upstream promoter transcription factor II (COUP-TFII) activates its expression [51]. However, it is insufficient to only control PEA-15 expression, and the phosphorylation must also be strictly regulated [52]. PEA-15 exists *in vivo* in three phosphorylation states: N – unphosphorylated at both Ser^104^ and Ser^116^, Pa – monophosphorylated at either Ser^104^ (Pa2) or Ser^116^ (Pa1), and Pb – diphosphorylated at both Ser^104^ and Ser^116^. To emphasize the critical importance of the phosphorylation states of PEA-15, which should remain relatively stable and consistent in order to perform its protective roles in specific cell types or tissues, we call the dynamic balance of different phosphorylation states of PEA-15 as “phosphorylation homeostasis” (Figure 1).

The phosphorylation states of PEA-15 play significantly greater roles in regulating cellular functions than does its expression level, and many conditions only affect the phosphorylation states, but not the total protein expression level. In opiate abusers, Ser^116^ phosphorylated PEA-15 in the prefrontal cortex decreases compared with age-, gender-, and postmortem delay (PMD)-matched controls, but not total PEA-15 level [53]. Similar results were reported from a study of morphine treated rats, in which repeated morphine treatment and opiate withdrawal did not modulate total PEA-15 content in the brain, but p-Ser^116^ PEA-15 was significantly up-regulated in the striatum and cortex following three days of opiate withdrawal, in accordance with elevated activation of Akt1 [54]. Cocaine treated rats did not alter the total level of PEA-15 in the cerebral cortex [55]. The PEA-15 gene expression did not display any significant difference in schizophrenia patients comparing to control subjects [56].

The roles of protein kinases in regulating PEA-15 functions have been well documented. Evidences implied the involvement of Ser^104^ phosphorylation of PEA-15 in the impairment of glucose metabolism [6,8]. It has been demonstrated that doubly phosphorylated PEA-15 (Pb) abrogates its ability to prevent nuclear translocation of ERK1/2 *in vivo* and *in vitro* [57]. It was further demonstrated that phosphorylation at Ser^104^ blocks ERK binding, and Ser^116^ phosphorylation promotes recruitment of PEA-15 into the DISC, inhibiting apoptosis [58]. Phosphorylation of PEA-15 seems to switch PEA-15 from a tumor-suppressor to a tumor-promoter [59]. Up-regulation of Akt in breast cancer cells suggests that phosphorylation of PEA-15 Ser^116^ could represent a key molecular mechanism in the resistance to chemotherapy in breast cancer patients [35]. Akt overexpression stabilizes endogenous PEA-15 and increases its half-life [60]. Phosphorylation of PEA-15 at Ser^116^ is highly abundant in astrocytomas and glioblastomas, which renders glioma cells resistant to glucose deprivation-mediated cell death [61]. The phosphorylation of PEA-15 is required for activation of JNK and inducing glioma cell autophagy [42]. AMP-activated protein kinase (AMPK), normally activated in both normal and cancer cells under stresses, such as nutrient deprivation, hypoxia, oxidative stress, or endoplasmic reticulum (ER) stress [62], can directly phosphorylate PEA-15 at Ser^116^ in primary human mammary epithelial cells (HMECs), promoting anti-apoptotic function through inhibiting DISC formation [63]. AMPK signaling plays important roles in anoikis (a form of apoptosis that is induced by anchorage-dependent cells detaching from the surrounding extracellular matrix) resistance under metabolic stress conditions, and the action of AMPK-PEA-15 is required for mammosphere formation and the anchorage independent growth of breast cancer cells [63].

Indirect mechanisms could also affect PEA-15 phosphorylation state. The 67 kD laminin receptor (67LR), the non-integrin cell-surface receptor for the extracellular matrix (ECM) formed by dimerization of the 37 kD cytosolic precursors (37LRP), is highly expressed in human cancers and widely recognized as a molecular marker of metastatic aggressiveness [64]. PEA-15 was found to interact with 67LR in both PED/PEA-15-transfected HEK-293 cells and in U-373 glioblastoma cells [65]. PEA-15 overexpression in HEK-293 cells increases 67LR-mediated cell adhesion and migration to laminins, which in turn activates PKC and CaMKII, causing doubly phosphorylation of PEA-15 [65]. The change of phosphorylation state of PEA-15 through interaction with 67LR induces cell responses to ECM-derived signals for cell survival in a poor microenvironment by enhancing cell proliferation and resisting apoptosis, favoring metastatic spread and colonization. Another mechanism affecting phosphorylation homeostasis involves chaperone-mediated autophagy (CMA). Non-phosphorylated, tumor suppressing form of PEA-15 seems to be more susceptible to CMA, by which non-phosphorylated PEA-15 is preferentially targeted by chaperone protein, Hsc70, and transported across the lysosomal membrane for degradation, while doubly phosphorylated PEA-15 is resistant to CMA. The hallmark of upregulated CMA activity in most cancer types enhances oncogenesis by shifting the balance of PEA-15 phosphorylation homeostasis toward tumor promotion [66].

Serine/threonine protein phosphatases (PPs) play equally important roles as protein kinases in regulating phosphorylation homeostasis of PEA-15. However, PEA-15 specific cytosolic PPs are much less understood comparing to kinase counterparts. Early evidence suggested that protein phosphatase 2A (PP2A) dephosphorylates PKC product of PEA-15 (p-Ser^104^) in astrocytes [1]. A recent study also linked PP2A and PEA-15 in protecting neurons against permanent and focal ischemic brain damage [67]. Tumor suppressor PTEN (phosphatase and tensin homolog on chromosome 10) [68] is a phosphatase that has been associated with reduced phosphorylation at Ser^116^ of PEA-15 [69]. PTEN loss-of-function mutations, commonly seen in tumor cells, result in increased phosphorylation at Ser^116^ of PEA-15, and enhancing PEA-15 binding to FADD, inhibiting Fas-induced apoptosis [70]. PTEN activation and PEA-15 dephosphorylation promote Fas-induced apoptosis by releasing FADD, and suppress ERK dependent proliferation in cancer cells [71]. PTEN seems to modulate p-Ser^116^ content through mediating Akt activity, but not through direct dephosphorylation of PEA-15. Another ubiquitously conserved serine/threonine phosphatase, PP4 [72], strongly affects phosphorylation at Ser^116^ of PEA-15, through which PP4 regulates apoptosis and proliferation of human cancer cells [73]. Overexpression of PP4 correlates with dephosphorylation of p-Ser^116^, with increased apoptosis and reduced proliferation, while PP4 knockdown results in significant elevation of p-Ser^116^ content, stimulating proliferation and inhibiting apoptosis. PP4 has also been demonstrated to regulate apoptosis of T-cells in human leukemia and lymphoma, and PP4-induced leukemic T-cell apoptosis is mediated through dephosphylation of p-Ser^116^ of PEA-15 [74].

Changes in PEA-15 phosphorylation states could fundamentally modulate PEA-15 functions and could initiate distinct mechanisms. In H-Ras transformed kidney epithelial cells, PEA-15 does not inhibit H-Ras-activated proliferation, while it seems to promote G_1_- to S-phase transition [75]. In these cells, PEA-15 appears to be phosphorylated at Ser^116^, which does not sequester ERK in the cytoplasm when co-expressed with constitutively active H-Ras. PEA-15 co-localizes with PLD1 in the nuclear and cytoplasmic regions surrounding the nucleus [75]. Through the interaction with PLD1, PEA-15 activates PKCα and ERK, which subsequently increases CDK4 and cyclin D, accelerating G_1_/S cell cycle transition [75]. The roles of PEA-15 in enhancing or impairing tumorigenesis depend on active signaling pathways and phosphorylation homeostasis in specific tumor cells.

As the expression level of PEA-15 is generally consistent in many cell types and tissues, the PEA-15 functions are mainly regulated through phosphorylation homeostasis. This homeostasis is mediated through the combined effects of protein kinases and protein phosphatases, involving crosstalk and interactions among various signaling pathways, including MAPK, Akt, and apoptosis. Loss-of-function mutations in tumor suppressors, such as PTEN and PP4, and over-activation of protein kinases, such as Akt and PKC, identified in many cancer types, could significantly modulate PEA-15 homeostasis in cells.

## 4. Structural Basis of PEA-15 Interactions

PEA-15 structure was first determined by solution NMR spectroscopy more than a decade ago [19], and the DED structure was further refined to high definition with detailed polar side chain interactions [22]. However, its interactions with other proteins have still been largely mysterious. Among major interactions PEA-15 is involved, the PEA-15/ERK2 interaction has been studied most. PEA-15 binds to ERK1 and ERK2 tightly with *K*_d_ (dissociation constant) in sub-micromolar (μM) range, and phosphorylation states of either PEA-15 or ERK do not affect the stability of the complex [76]. This seems to be contradictory to the switched specificity from ERK to FADD upon PEA-15 phosphorylation [58]. Furthermore, ERK2 is in its monomeric state when binding to PEA-15 at 1:1 ratio [77]. Early NMR evidence showed that residues from both DED, such as the charge-triad residue D^74^, and the C-terminal tail of the PEA-15 were necessary for ERK2 binding [19]. It was further demonstrated that PEA-15 C-terminal tail contained a reversed binding sequence, ^121^IKLAPPPKK^129^, of ERK2 D-recruitment site (DRS) [26,76]. However, the role of DED residues, particularly D^74^, have not been fully defined, although the D^74^A mutation abolishes the binding capacity of PEA-15 to ERK2 [19]. Our NMR dynamic study suggested that D^74^ is not at the binding interface between PEA-15 and ERK2, and PEA-15 utilizes its DED residues from helices α1, α5, and α6 to bind ERK2 [26]. This finding was later confirmed by a crystallographic study of the complex, which illustrated that the binding interface involves residues D^19^, E^68^, R^71^, P^73^, L^76^, and V^80^ from PEA-15, while the charge-triad residues R^72^-D^74^L^75^ are not at involved in direct interaction with ERK2 [25]. Upon ERK2 binding, PEA-15 DED undergoes substantial conformational change, particularly at the helices α2, α3, and α4 [26,27]. Both NMR relaxation experiments [26] and PEA-15/ERK2 crystal structures [25] suggested a disordered region around helix α3 in the complex. A plausible allosteric binding model suggested that upon recognition of ERK2 DRS by its C-terminal tail, PEA-15 induces significant conformational changes at DED to reveal binding interface to ERK2, and the conformational changes are mediated by the polar interactions on the DED surface, including the charge-triad hydrogen bonding network [26,27]. D74A mutation disrupts this crucial hydrogen bonding network, disabling the conformational flexibility necessary for ERK2 binding [27].

Another major PEA-15 interaction involves PLD isoforms 1 and 2, resulting in elevation of intracellular levels of diacylglycerol that activates diacylglycerol-sensitive PKCα isoform [7]. Activation of PKCα in PEA-15 overexpressing cells and tissues prevents insulin induction of the PKCζ isoform, which is the major activator of glucose transporter 4 (GLUT4) [8,78]. The PEA-15/PLD1 interaction mostly occurs between residues 762-801 of the C-terminal domain, termed D4α, of PLD1 [79], and the first 24 residues of the PEA-15 [28]. PEA-15 binding interface to PLD1-D4α are mostly located in α1, α3, α4 helices and α1-α2 and α3-α4 loop, while helix α5 seems to play a role in allosteric regulation of the binding [29]. The binding interfaces of PEA-15 to ERK2 and PLD1 are partially overlapping, consistent with mutually exclusive binding modes between ERK2 and PLD1, and the DED conformational flexibility may play a role in recognition of various binding partners.

**Figure 2:**
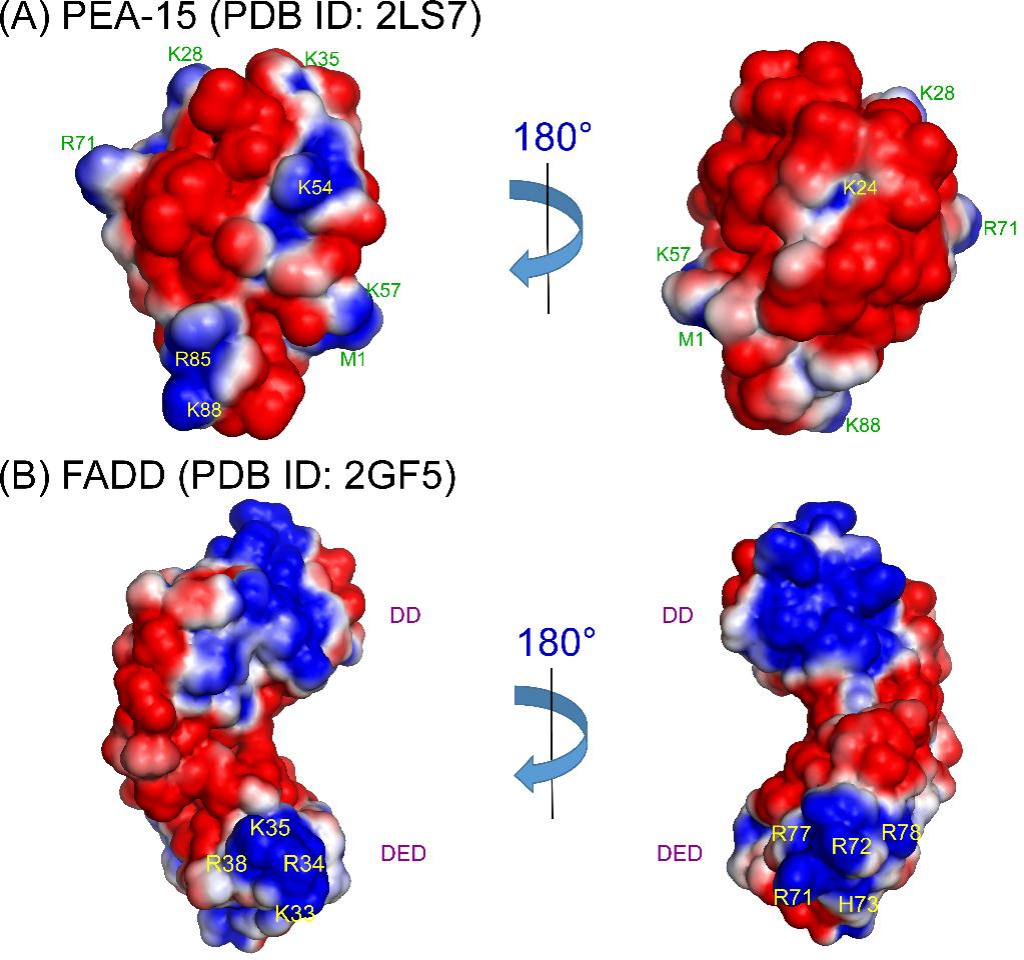
Surface electrostatics of (**a**) PEA-15 (PDB ID: 2LS7) DED and (**b**) FADD (PDB ID: 2GF5) DED. (**a**) PEA-15 DED possesses two distinctive surfaces with one surface consisting of positive/negative patches, and the other surface consisting a more continuous negative patch with a positively charged pocket around K^24^, which could provide the self-association site for p-Ser^104^/p-Ser^116^. (**b**) FADD DED possesses two positive patches on opposite surfaces, which could provide binding interface to PEA-15 negative patch on DED and/or C-terminal phosphoryl groups. Surface electrostatics was calculated using APBS 1.4.1 [94] and plotted using PyMOL 1.7.2 [95]. Positive charges are shown in blue with residues labeled, and negative charges are in red.

The homotypic DED-DED interactions between PEA-15 and FADD or procaspase-8 are largely unexplored in terms of structural characterizations. DEDs are highly flexible domains, and are fairly difficult to crystalize. Currently, only a viral FLICE-like inhibitory protein (vFLIP), MC159 [80,81], and PEA-15/ERK2 complexes [25] have been crystalized in DED subfamily, and there are no structures, crystal or NMR, of homotypic DED-DED complexes up to date (although the vFLIP contains tandem DEDs). Based on complex structures in DD and CARD subfamilies, the homotypic complex formation is most likely mediated by electrostatic interactions between oppositely charged surface patches between the two domains [21]. For PEA-15 to involve in homotypic interactions with FADD or procaspase-8 in the DISC, phosphorylation at Ser^116^ is crucial [58]. Here we suggest two possible binding models for PEA-15/FADD homotypic interactions: (1) PEA-15 C-terminal tail phosphorylation may cause self-association of the negative phosphoryl group with a positive patch on the DED to form extended negative surface on the DED, promoting the electrostatic interactions with positive surface patches on FADD DED; (2) PEA-15 C-terminal tail phosphorylation may recognize a separate positively charged surface on FADD DED, promoting additional electrostatic interactions between FADD and PEA-15 DEDs. We have examined the surface electrostatics of PEA-15 (PDB ID: 2LS7) [22] and full-length FADD (PDB ID: 2GF5) [82] (Figure 2). PEA-15 DED shows two distinct surface patterns. On one surface, it possesses both positive and negative patches, but on the opposite side, the DED surface is almost all negatively charged, formed by helix α1, α1-α2 loop, dynamic helix α3, α3-α4 loop, and α5-α6 loop, with a small positively charged pocket surrounding K^24^ on helix α2 (Figure 2A). In model 1, the phosphorylated C-terminal tail may self-associate with this K^24^ pocket to form a uniform negative surface to promote DED-DED interaction. On the FADD DED, there are two distinct positive surface patches, one formed by K^33^, R^34^, K^35^, and R^38^ along helix α3, and the other one formed on the opposite side by R^71^ on α5-α6 loop, and R^72^, H^73^, R^77^, and R^78^ on helix α6 (Figure 2B). In model 2, C-terminal tail phosphorylation on PEA-15 may recognize one of the positive surface patches on FADD DED, promoting binding of the negative surface of PEA-15 DED to the second positive patch of FADD DED. This binding model is similar to PEA-15/ERK2 binding, where the C-terminal tail of PEA-15 recognizes ERK2 DRS, promoting association of PEA-15 DED to ERK2 as discussed earlier [25,26,76]. The fact that phosphorylation of PEA-15 C-terminal tail does not affect ERK2 binding affinity [76] suggests that PEA-15/FADD binding be more likely to follow model 2 because this model does not change PEA-15 DED surface features or dynamic properties, but model 1 cannot be completely excluded. The conformational flexibility of PEA-15 DED, particularly at regions around helix α3, may also play a role in the homotypic interactions. A similar conformational flexibility around helix α3 has also been reported for PYD [21,83]. These binding models still await for the confirmation from actual DED-DED complex structures.

## 5. Therapeutic Interventions Involving PEA-15

As PEA-15 has been heavily implicated in various diseases with both detrimental and beneficial effects, therapeutic strategies can be developed targeting PEA-15 and its phosphorylation homeostasis. Recently Greig and Nixon reviewed the involvement of PEA-15 in various diseases, such as cancer, type 2 diabetes, neurological disorders, and cardiovascular disease [18], and concluded the pivotally important roles of PEA-15 in regulating diverse cellular processes and the potential to target PEA-15 therapeutically for many diseases. In this section, we focus our discussion on the actual therapeutic interventions and utilizations of PEA-15 in cancers, with emphasis on the importance of phosphorylation homeostasis.

PEA-15 is an endogenous protein that has been clinically proven to be beneficial to patients of various cancers [38,39,40]. The beneficial effects are generally associated with the dephosphorylated form of the protein. For clinical applications, the serine residues at the two phosphorylation sites of PEA-15 are commonly mutated into either an alanine (A) to represent the non-phosphorylated state, or an aspartic acid (D) to mimic phosphorylated state. Using a human ovarian cancer tissue microarray, Lee *et al.* demonstrated that ovarian cancer tissues express significantly more doubly phosphorylated PEA-15 than do adjacent normal tissues [84]. In addition, nonphosphorylatable PEA-15 S^104^A/S^116^A mutant (PEA-15-AA) exerts a more potent inhibition of tumor cell migration and *in vivo* angiogenesis than does phosphomimetic S^104^D/S^116^D mutant (PEA-15-DD) [84]. Another study showed that knockdown of PEA-15 in ovarian cancer cells resulted in resistance to paclitaxel, a standard chemotherapeutic agent for ovarian cancer [85]. Overexpressing PEA-15-DD, the bis-phosphorylated PEA-15 mimic, in ovarian cancer cell lines greatly enhances paclitaxel sensitivity with reduced cell viability and anchorage-independent growth [85]. The mechanism of PEA-15-mediated paclitaxel sensitization involves impairing functions of SCLIP (superior cervical ganglion-10-like protein), a microtubule dynamics regulatory protein, blocking microtubule destabilization, and promoting mitotic arrest and apoptosis [85]. Clear cell carcinoma (CCC) of the ovary renders chemoresistant through epidermal growth factor receptor (EGFR) and the downstream targets of MEK/ERK pathway. Both EGFR tyrosine kinase inhibitor, erlotinib, and MEK inhibitor, selumetinib (AZD6244), significantly suppress tumor growth and induce G_1_ arrest in a CCC xenograft model in a PEA-15 dependent manner, and knockdown of PEA-15 results in selumetinib-resistant cells [86]. In addition, cells transfected with PEA-15-AD, mimicking mono-phosphorylation at S^116^, are more sensitive to selumetinib than those transfected with PEA-15-AA [86]. PEA-15 can also promote ER-anchored protein tyrosine phosphatase PTP1B functions to dephosphorylate EGFR in triple negative breast cancer (TNBC) cells, down-regulating EGFR signaling in cancer cells [87].

As general strategies for PEA-15 targeted therapy, three major approaches can be considered: (1) inhibiting protein kinase activities, including PKC, CaMKII, and Akt; (2) enhancing tumor suppressor/phosphatase activities, particularly PTEN and PP4; (3) intervening protein-protein interactions between PEA-15 and FADD, ERK, or PLD. As presented earlier, PEA-15, at the crossroad of several major signaling pathways, may play determinant roles of cell fate, death or proliferation, through modulating its phosphorylation homeostasis. In the CNS, most PEA-15 should be in its phosphorylated state to prevent cell death of irreplaceable neurons, while in peripheral cells and tissues, PEA-15 should mostly remain unphosphorylated to prevent harmful proliferation and to sensitize malignant cells to apoptosis. In inflammatory and cancerous conditions, however, the kinase activities, such as Akt, are greatly enhanced, while the tumor suppressing phosphatases, such as PTEN, are heavily mutated to loss-of-function. This unbalanced kinase/phosphatase activities cause significant increase in phosphorylated contents of PEA-15, lead to uncontrolled MAPK/ERK-dependent malicious cell proliferation and resistance to chemotherapy-induced cancer cell death. Some Akt inhibitors have undergone clinical trials to treat neuroblastoma [88], a condition related to PEA-15 phosphorylation, but without much success. There have been efforts to target MAPK pathway in cancer treatment [89,90]. However, targeting MAPK pathway often suffers from high toxicity and low efficacy due to a general network motif, termed incoherent feed-forward loop [91]. PEA-15 interactions with FADD and procaspase-8 can also be targeted to induced apoptosis of cancer cells. However, the lack of structural information on the protein-protein interactions limits our ability to develop drugs that target the interface of the proteins. The DED conformational flexibility should also be considered when targeting protein-protein interactions involving PEA-15. Similarly, PEA-15/PLD interactions may be targeted to reduce insulin resistance. Another approach could be promotion of dephosphorylation of PEA-15, which requires enhanced activity or induced overexpression of related phosphatases, whose expression is commonly suppressed in many cancer types.

There were also efforts to develop PEA-15 protein itself as therapeutic agents. As discussed earlier, PEP-1-PEA-15 can be potentially developed as novel protein therapeutic strategy for treatment of a variety of neurodegenerative diseases, such as PD, utilizing its anti-apoptotic and neuroprotective functions [33]. In advanced breast cancer treatment, PEA-15 was targeted by a breast cancer construct, T-VISA, composed of the human telomerase reverse transcriptase (hTERT; T) promoter and a versatile transgene amplification vector VISA (VP16-GAL4-WPRE integrated systemic amplifier). T-VISA is a robust cancer-specific protomer, which specifically drives target gene expression in breast cancer cells with high efficiency [92]. It has been shown that T-VISA-PEA-15 selectively and significantly suppressed proliferation of several breast cancer cell lines and induced apoptosis in primary breast cancer cells, while normal breast cells were largely not affected [93]. Additionally, T-VISA-PEA-15 nanoparticle treatment in the breast tumor mouse model demonstrated effective suppression of tumor growth and prolonged survival, with virtually no acute toxicity [93].

In summary, PEA-15 is a small protein without any catalytic activity. Nevertheless, it has been identified to regulate several crucial cellular pathways that determine the fate of the cells. It possesses high potential therapeutic value, and has been demonstrated to be vital in clinical treatment of cancer patients. As an endogenous protein, PEA-15 based therapeutics would not expect to exert severe side effects or high toxicity, and therefore, could be of great potential for treatments of cancers and neurodegenerative diseases.
